# “External validation of the LENT and PROMISE prognostic scores for malignant pleural effusion” Craig A. Mounsey, Nikolaos I. Kanellakis, Dinesh N. Addala, Jamie A. Mawhinney, Nick Freemantle and Najib M. Rahman. *ERJ Open Res* 2025; 11: 01019-2024.

**DOI:** 10.1183/23120541.51019-2024

**Published:** 2025-07-21

This article was originally published without a support statement. The support statement is shown below. This has also been added to the article itself.

Support statement: This work was supported by the Chinese Academy of Medical Sciences (CAMS) Innovation Fund for Medical Science (CIFMS), China (grant number 2024-I2M-2- 001-1). N.I. Kanellakis is supported by Asthma + Lung UK grant RJF25\31.

